# Exploratory Evaluation of Quantitative Rubidium-82 PET Myocardial Perfusion Parameters: Absolute Blood Flow, Flow Reserve, and Left Ventricular Function in an Arabian Gulf Cohort

**DOI:** 10.3390/jcm15187059

**Published:** 2026-09-11

**Authors:** Ahmad Alenezi, Masoud Garashi, Satish Panchadar, Gautam Biswas

**Affiliations:** 1 Department of Radiologic Sciences, Faculty of Allied Health Sciences, Kuwait University, Kuwait City 13110, Kuwait; 2 Nuclear Medicine Department, Kuwait Chest Disease Hospital, Kuwait City 13110, Kuwait

**Keywords:** rubidium-82, PET, myocardial blood flow, myocardial flow reserve, exploratory analysis, left ventricular function, Arabian Gulf, Kuwait

## Abstract

**Background/Objectives**: Rubidium-82 (^82^Rb) PET myocardial perfusion imaging (MPI) yields, in a single study, quantitative absolute myocardial blood flow (MBF, mL/min/g), myocardial flow reserve (MFR), and gated left ventricular (LV) function (LVEF, EDV, ESV, SV). These quantitative values depend on the tracer, scanner, kinetic model, software, and underlying population and have been characterised almost exclusively in North American and European cohorts. The Arabian Gulf, where Kuwait has among the highest age-standardised diabetes prevalence worldwide (25.6%), is essentially unstudied, so the distribution and behaviour of these parameters in such a real-world cardiometabolic population are unknown. To evaluate the distribution of the quantitative ^82^Rb PET parameter set in a real-world Arabian Gulf cohort and, within a small clinically defined normal subgroup, to describe sex- and age-related patterns in absolute MBF, MFR, and LV function. Given the limited size of the normal subgroup, these values are presented as exploratory, hypothesis-generating observations rather than definitive population reference norms. **Methods**: Retrospective single-centre study of 330 consecutive ^82^Rb PET/CT studies (analytic cohort *n* = 292 after exclusion of repeat studies and those with incomplete quantitative output; mean age 63.1 ± 12.1 years) who underwent adenosine-stress ^82^Rb PET/CT MPI. The clinically defined normal subgroup, normal perfusion (C1), normal global MFR (≥2.0), normal resting LVEF, and no documented cardiac history, comprised 44 patients (25 female, 19 male). Reference values are reported as sex- and age-stratified centiles (5th, 25th, median, 95th percentiles), with the 5th percentile reported for each parameter. Sex differences used Mann–Whitney U with rank-biserial r and Cohen’s d (95% CI); age was examined across broad bands. All analyses followed APA 7 standards with Bonferroni correction. **Results**: In the clinically defined normal subgroup, median global stress MBF was 2.94 mL/min/g (5th percentile 2.03) and median global MFR was 2.72 (5th percentile 2.06); every value satisfied the C1 definition (MFR ≥ 2.0) by construction, so these limits describe the preselected subgroup and cannot independently validate the 2.0 threshold. Women had higher resting MBF than men (median 1.07 vs. 0.90 mL/min/g; *p* = 0.007), with numerically lower global MFR that did not survive correction for multiple comparisons (2.57 vs. 2.98; *p* = 0.022); stress MBF did not differ by sex (3.00 vs. 2.91; *p* = 0.522). LV volumes were smaller in women, while LVEF was similar between sexes. The 5th percentile for stress LVEF was 54% (women) and 53% (men). These absolute-flow reference values are lower than those reported for Western low-risk cohorts (stress MBF ~3.25 mL/min/g; MFR ~3.18), consistent with the higher cardiometabolic burden of this population. **Conclusions**: In this exploratory single-centre evaluation, quantitative ^82^Rb PET flow values in a small clinically defined normal Arabian Gulf subgroup were lower than Caucasian-derived values, while sex-related differences in resting flow were evident and age-related differences were numerically consistent with previously reported trends. Because the normal subgroup is small, these findings are hypothesis-generating and require confirmation in larger, prospectively screened cohorts before use as population reference values; they nonetheless indicate that population- and pipeline-specific calibration is needed when quantitative ^82^Rb thresholds derived elsewhere are applied locally.

## 1. Introduction

Quantitative positron emission tomography (PET) measures absolute myocardial blood flow (MBF) and myocardial flow reserve (MFR) in physiological units (mL/min/g), adding information on coronary physiology beyond relative perfusion [1]. These estimates have been validated against nitrogen-13 ammonia [2] and oxygen-15 water [3], and professional-society guidance now recommends incorporating MBF and MFR into standard stress PET reporting [4,5], with recent guidance extending quantitative flow to newer tracers [6]. Rubidium-82 (^82^Rb), a generator-produced tracer requiring no on-site cyclotron [7], is the most widely used PET perfusion agent, and its absolute quantification depends on a robust kinetic-modelling pipeline [8,9]. The clinical value of quantitative flow lies in what relative perfusion imaging cannot show. Relative images depict the distribution of tracer uptake and therefore identify regional differences, but they are normalised to the segment of highest uptake and can appear normal when flow is reduced uniformly across the myocardium. Balanced multivessel disease and diffuse microvascular dysfunction both produce this pattern. Absolute quantification resolves the ambiguity by expressing flow in physiological units, so that a globally reduced hyperaemic response is measurable even when the relative images are unremarkable. Reduced flow reserve identified in this way carries independent prognostic weight, and its incorporation into routine reporting has been endorsed on that basis [4,5]. This diagnostic gain, however, is contingent on the availability of a reference distribution appropriate to the patient being scanned, since the interpretation of an absolute value depends entirely on what is considered normal.

A defining feature of these quantitative values is that they are not universal. Absolute flow varies with tracer, scanner, stress agent, kinetic model, and software, as shown in the multi-software RUBY-10 comparison [10], and reference behaviour also varies with age and sex [11,12] and with the metabolic profile of the population studied. In that comparison, absolute flow values derived from identical dynamic datasets differed materially according to the software platform and kinetic model applied, establishing that a quantitative threshold is a property of a processing pipeline as much as of a patient. Subsequent multicentre and single-centre European series have reported reference distributions that differ from North American values in both central tendency and spread [13,14,15], reinforcing the point that no single set of limits can be assumed to transfer between institutions. Beyond these technical factors, the physiological determinants of hyperaemic flow, including endothelial function and myocardial oxidative metabolism, differ between populations [16]. Existing quantitative ^82^Rb characterisations derive almost entirely from North American and European cohorts; because diabetes and obesity impair microvascular function and lower baseline flow [17,18], values from those cohorts may not transfer to populations with a markedly different cardiometabolic burden.

The Arabian Gulf carries among the highest age-standardised burdens of diabetes and obesity worldwide (Kuwait 25.6% in 2024 [19]), yet the distribution and behaviour of quantitative ^82^Rb PET parameters in this setting have not been described. We therefore undertook an exploratory evaluation of the full quantitative ^82^Rb PET parameter set, MBF and MFR by territory, LVEF, and volumes, in a real-world Kuwaiti cohort using a single Corridor4DM quantification pipeline. Within a small, clinically defined normal subgroup we additionally describe sex- and age-related patterns; because that subgroup is limited in size, these are presented as exploratory, hypothesis-generating observations rather than definitive population reference norms. To our knowledge, this is the first study to report sex- and age-stratified quantitative ^82^Rb PET reference estimates for an Arabian Gulf population.

## 2. Materials and Methods

### 2.1. Study Design and Patient Selection

This retrospective single-centre study was approved by the institutional ethics committee with a waiver of informed consent for the use of anonymised data (approval no. 2465). Of 330 consecutive ^82^Rb-chloride PET/CT myocardial perfusion studies (one-day gated rest-stress protocol with low-dose CT for attenuation correction) performed at Kuwait Chest Disease Hospital between January 2018 and December 2023, repeat studies in the same patient and studies with incomplete or unverifiable quantitative flow output were excluded, leaving an analytic cohort of 292 (Table 1). The flow of studies was therefore: 330 consecutive ^82^Rb PET/CT studies were screened; 38 were excluded as repeat studies in the same patient or for incomplete or unverifiable quantitative output; 292 studies formed the analytic cohort; and, within this, 44 studies met the criteria for the clinically defined normal subgroup. All values in Table 1 refer to the analytic cohort of 292 unless stated otherwise. Rubidium-82 chloride was produced using a CardioGen-82^®^ strontium-82/rubidium-82 generator (Bracco Diagnostics Inc., Princeton, NJ, USA) and delivered intravenously with its dedicated infusion system; pharmacologic stress was induced with adenosine. Patients were classified using the PET reporting C-category scheme. Studies were categorised C1–C7 by combining visual perfusion assessment with global flow reserve: C1 normal perfusion with MFR ≥ 2.0, C2 abnormal perfusion with MFR < 2.0, C3 normal perfusion with MFR < 2.0, C4 abnormal perfusion with MFR ≥ 2.0, and C5–C7 abnormal perfusion with abnormal reserve in one, two or three territories. The clinically defined normal subgroup, normal perfusion (C1), normal global MFR (≥2.0), normal resting LVEF, and no documented cardiac history, comprised 44 patients (25 female, 19 male). Patients in category C3 (normal perfusion with reduced MFR) were excluded from the normal reference because MFR is abnormal by definition in that group. This ^82^Rb PET cohort is the subject of several companion analyses addressing distinct questions: the association of reduced flow reserve with all-cause mortality; the detection of prior cardiac disease by absolute stress flow in normal-appearing studies; and PET–SPECT left-ventricular functional discordance. The present analysis is confined to the derivation of sex- and age-stratified reference estimates within a clinically defined normal subgroup and does not duplicate those analyses.

### 2.2. Image Acquisition

PET/CT: Rest and stress ^82^Rb PET/CT myocardial perfusion imaging was performed on a dedicated PET/CT scanner. A CT scout was acquired (kV 120, mA 10), followed by a helical CTAC scan (kV 120, mA 145, slice thickness 3.75 mm, pitch 0.531:1, rotation time 0.8 s, CTDIvol 19.85 mGy, DLP 340.75 mGy·cm). Pharmacologic stress was induced with adenosine (0.14 mg/kg/min over 6 min; caffeine and theophylline withheld ≥ 48 h). ^82^Rb chloride was administered from a CardioGen-82^®^ generator (target approximately 1110 MBq). Dynamic list-mode data were acquired for 7–8 min with ECG gating during the static phase (8 or 16 frames/cycle) over a single bed position (S100.0 to I50.4), with a 2 min pre-scan delay followed by a 4 min static emission acquisition. Reconstruction used OSEM (3 iterations, 18 subsets) with time-of-flight and point-spread-function modelling (Q.AC wide-view, VPFX-S; DFOV 70 cm, matrix 128 × 128, voxel size 2.0 mm).

### 2.3. Reference (Clinically Defined Normal) Subgroup Definition

The reference subgroup comprised PET C1 studies (visually normal stress and rest perfusion with global MFR ≥ 2.0) with documented absence of cardiac history, resting LVEF ≥ 50%, and age ≥ 18 years (*n* = 44). Categories were assigned clinically by the reporting physician at the time of the original report. For the present analysis, category assignment was re-derived from the quantitative output to ensure internal consistency between the label and the measured values. In one study the clinical label (C1) and the recomputed global MFR (1.25) disagreed; that study was excluded from the reference subgroup because it did not satisfy the stated MFR criterion. One paediatric study was excluded by the age criterion. No other discrepancy between the clinical label and the recomputed value was identified within the C1 stratum. All reference analyses are restricted to this subgroup; the analytic cohort of 292 studies is used only for cohort characterisation in Table 1.

### 2.4. Image Analysis

All myocardial perfusion datasets were processed with Corridor4DM (INVIA, Ann Arbor, MI, USA, version 2022), providing a unified quantification framework across all studies. For ^82^Rb PET, dynamic list-mode data were reframed into the standard acquisition sequence, and absolute myocardial blood flow (MBF, mL·min^−1^·g^−1^) was computed using a one-tissue-compartment kinetic model with dual spillover correction applied to the left-ventricular blood pool and right-ventricular input functions. Myocardial flow reserve (MFR) was derived as the ratio of stress to rest MBF, globally and for the LAD, LCx, and RCA territories defined on the standardised 17-segment model. For gated studies, 4DM automatically delineated left-ventricular endocardial and epicardial surfaces and computed LVEF, EDV, ESV, and stroke volume at rest and stress. All automatically generated contours were visually inspected by an experienced operator and manually corrected where necessary. Because absolute flow and functional values depend on the kinetic model and software platform, a single quantification pipeline was used throughout; derived reference values are therefore specific to this Corridor4DM^®^ implementation.

Myocardial blood flow was derived from the dynamic first-pass data using the one-tissue-compartment model implemented in the software, with arterial input sampled from a region placed in the left atrial or left ventricular blood pool and corrections applied for partial-volume effect and spillover. Flow was computed for each of the three principal vascular territories and as a global value, at rest and at peak hyperaemia. Myocardial flow reserve was calculated as the ratio of hyperaemic to resting flow for each territory and globally. Gated data were processed separately at rest and after stress to yield ejection fraction, end-diastolic volume and end-systolic volume; stroke volume was derived as the difference between end-diastolic and end-systolic volume. All studies were processed by the same operator using identical software settings, and each dataset was reviewed for adequacy of myocardial contour placement, for arterial input curve quality, and for patient motion between the dynamic and gated acquisitions; studies with uncorrectable motion or with an implausible input function were excluded before analysis. Because absolute values are pipeline-dependent, the reference estimates reported here should be regarded as specific to this acquisition and quantification protocol.

### 2.5. Statistical Analysis

Analyses were performed in R (v4.3.x) following APA 7 standards with exact *p*-values to three decimal places and 95% confidence intervals for effect sizes. Reference values are reported as centiles (5th, 25th, median, 95th percentiles), with the 5th percentile reported for each parameter, overall and stratified by sex and by broad age band. Sex differences used Mann–Whitney U with rank-biserial r and Cohen’s d; age effects were examined by Kruskal–Wallis H. Bonferroni correction was applied across the eight comparisons. Reference limits are reported as exploratory estimates with bootstrap percentile 95% confidence intervals (2000 resamples); given subgroup sizes of 19–25, sex- and age-specific limits are unstable and should be interpreted as provisional.

Effect sizes are reported as Cohen’s d with pooled standard deviation and 95% confidence intervals; positive values indicate higher values in men.

## 3. Results

### 3.1. Cohort Characteristics and Normality

Cohort characteristics for the full PET cohort and the clinically defined normal reference subgroup are summarised in Table 1. Consistent with the retrospective clinical sampling, distributions of the quantitative parameters departed from normality (Shapiro–Wilk), supporting the use of non-parametric, centile-based reference statistics throughout.

### 3.2. MBF and MFR Reference Values (PET)

Median global stress MBF in the clinically defined normal subgroup was 2.94 mL/min/g (5th percentile 2.03, 95th percentile 3.69) and median global MFR was 2.72 (5th percentile 2.06, 95th percentile 4.00). By definition every patient satisfied MFR ≥ 2.0; the 5th percentiles therefore sit immediately above the conventional threshold by construction, and are descriptive of the preselected subgroup rather than independent confirmation of the threshold (Table 2).

Territorial stress MBF showed the expected regional pattern, with the RCA territory demonstrating the highest median value (3.26 mL/min/g) and the LAD the lowest (2.65 mL/min/g); this distribution was consistent across age groups.

### 3.3. Sex-Stratified MBF and MFR Reference Values

Table 3 presents sex-stratified MBF and MFR reference values. Women had higher resting MBF than men (median 1.07 vs. 0.90 mL/min/g) and numerically lower global MFR (2.57 vs. 2.98), with stress MBF similar between sexes (3.00 vs. 2.91 mL/min/g), the higher resting flow in women accounting for the lower reserve, and the sex pattern consistently reported in published ^82^Rb PET reference series.

Formal between-sex comparisons within the clinically defined normal subgroup were performed using Mann–Whitney U tests with Bonferroni correction across six comparisons (α = 0.00833). Women had higher global resting MBF than men (median 1.07 vs. 0.90 mL/min/g; U = 124, *p* = 0.007, r = −0.48) and lower LAD flow reserve (2.35 vs. 3.15; U = 364, *p* = 0.002, r = +0.54); both differences survived correction. Global MFR (2.57 vs. 2.98; *p* = 0.022, r = +0.40) and LCX flow reserve (2.44 vs. 2.91; *p* = 0.045, r = +0.36) were nominally lower in women but did not survive correction. Global stress MBF did not differ by sex (3.00 vs. 2.91 mL/min/g; *p* = 0.522), nor did RCA flow reserve (*p* = 0.218).

### 3.4. Age-Stratified MBF and MFR Reference Values

Global MFR and stress MBF were numerically lower in the older stratum, but neither reached significance (stress MBF 3.11 vs. 2.66, *p* = 0.160, r = 0.25; MFR 2.87 vs. 2.59, *p* = 0.300, r = 0.19; rest MBF *p* = 0.687). The direction is concordant with the negative age correlation for MFR documented by Sperry et al. [12], although the present subgroup is too small to demonstrate it. Table 4 presents age-stratified MBF and MFR values.

### 3.5. LV Functional Parameters: Sex Differences

In the clinically defined normal subgroup, LV volumes differed by sex: men had larger LV volumes than women. Differences surviving Bonferroni correction were stress EDV (82 vs. 61 mL; r = 0.48; d = 0.88, 95% CI 0.25–1.50; *p* = 0.006), stress SV (52 vs. 41 mL; r = 0.56; d = 1.03, 0.39–1.67; *p* = 0.001) and rest SV (38 vs. 31 mL; r = 0.49; d = 0.78, 0.16–1.40; *p* = 0.005). Rest EDV and rest ESV differed nominally but did not survive correction. Neither stress nor rest LVEF differed by sex (both r ≈ 0.05, with confidence intervals for d spanning zero). Stress and rest LVEF did not differ significantly by sex within this stratum (stress median 67% vs. 67%; *p* = 0.81), consistent with the small, well-characterised sex-related differences in LV geometry on gated imaging [20,21]. The larger sex differences in volume than in ejection fraction reflect proportional scaling of end-diastolic and end-systolic volumes (Table 5).

### 3.6. Age Effects on LV Functional Parameters

Across broad age bands, absolute stress MBF and MFR declined modestly with age, while LVEF was comparatively age-stable, consistent with the expected age dependence of flow reserve and the relative age independence of ejection fraction. Given the size of the clinically defined normal subgroup, age-stratified centiles are reported for two broad bands and should be regarded as indicative (Table 6).

### 3.7. LV Functional Reference Values (By Sex)

Table 7 presents sex-stratified reference values for LV functional parameters in the clinically defined normal subgroup. The 5th percentiles for stress LVEF were 54% (women) and 53% (men), physiologically appropriate lower bounds for a normal reference, in contrast to the implausibly low values obtained before the stratum was restricted to clinically defined normal patients.

### 3.8. Age-Stratified LV Functional Reference Values

Table 8 presents age-stratified reference values for key LV functional parameters. As expected from the Kruskal–Wallis analysis, stress LVEF was stable across age groups (medians 55–68%), while stress SV declined in the older groups.

## 4. Discussion

### 4.1. Summary and Contributions

To our knowledge, this is the first study to provide an exploratory sex- and age-stratified reference dataset for the complete ^82^Rb PET parameter set reported in an Arabian Gulf population. No prior reference to the ^82^Rb PET series appears to have been derived from an Arabian Gulf or Middle Eastern cohort, as existing databases originate predominantly from North American and European populations [11]; this represents a population and geographic gap rather than a claim of absolute primacy, since a fully exhaustive search of regional and non-indexed literature was beyond the scope of this work.

The dataset’s value lies in several further features. It reports absolute MBF, MFR, and LV functional parameters from a single integrated cohort and quantification pipeline, in a population under-represented in existing ^82^Rb reference data, using categories re-derived from source data to ensure internal consistency with the C1 definition.

### 4.2. MBF and MFR: Clinical Context

The ASNC/SNMMI abnormal MFR threshold of 2.0 derives largely from predominantly Caucasian cohorts [4]. Because the reference subgroup was defined in part by global MFR ≥ 2.0, its MFR distribution is preselected by construction and cannot independently validate the 2.0 threshold. The 5th-percentile MFR of 2.06 (bootstrap 95% CI 2.03–2.20) is reported descriptively: it confirms that qualifying subjects cluster immediately above the entry criterion, as expected by design, and is not evidence for the threshold’s validity in this population. The broader clinical implication is methodological: lower reference limits are sensitive to the kinetic model, software platform, and cohort selection, so locally derived limits are preferable to transplanting limits from cohorts processed with different pipelines [10,18]. The territorial MFR pattern, with the highest reserve in the RCA territory, is consistent with regional variation reported in prior ^82^Rb PET reference data and supports territory-specific assessment alongside global MFR, as regional deficits may be obscured by global averaging. Placing our estimates alongside published series requires care, because the comparison is between pipelines as much as between populations. European quantitative Rb-82 series have themselves reported reference distributions that differ from North American values [13,14,15], and the RUBY-10 comparison demonstrated that this variation arises in part from the kinetic model and software applied to identical raw data [10]. Our global stress flow and flow reserve sit below the values reported in Western low-risk series, and the direction of that difference is consistent across territories rather than confined to one vascular distribution, which argues against a localised technical artefact. Whether the difference reflects the cardiometabolic profile of the population, the quantification pipeline, or the referral basis of the cohort cannot be resolved from these data, and we present it descriptively for that reason. The practical implication is not that one set of values is correct and another incorrect, but that a laboratory applying quantitative thresholds should establish what its own pipeline produces in patients it considers normal before adopting limits derived elsewhere.

### 4.3. Lower Absolute Flow and Its Metabolic Basis

Absolute stress MBF and MFR in this Gulf cohort were lower than values reported in Western low-risk PET series, a difference that may reflect the population’s high burden of diabetes and obesity, but which cannot be attributed to any single factor in this dataset. Differences from Western series may also arise from scanner, kinetic model, software, stress protocol and patient selection; no direct statistical comparison with external cohorts was performed, so this comparison is descriptive and the metabolic explanation is hypothesised rather than demonstrated. This matters clinically: quantitative thresholds and lower reference limits derived from predominantly Caucasian, metabolically healthier cohorts may not transfer directly, and applying them unmodified risks misclassifying flow as abnormal, or masking true impairment, in Gulf patients. Diabetic patients in particular show markedly reduced coronary flow reserve that carries prognostic weight even without epicardial disease [22], underscoring the need for population- and pipeline-specific calibration rather than a single universal cut-off.

### 4.4. Sex and Age Patterns

Within the normal subgroup, women had significantly higher resting MBF than men (*p* = 0.007) and significantly lower LAD flow reserve (*p* = 0.002); global MFR was numerically lower in women but did not survive correction, and stress MBF did not differ by sex. Flow was numerically lower in older patients without reaching significance, while ejection fraction remained comparatively stable. The higher perfusion in women is concordant with independent cross-modality evidence that female sex is associated with greater myocardial perfusion and blood volume beyond other physiological factors [23]. That the sex-related differences in resting flow are reproduced in an independent population and pipeline supports the validity of the measurements, and the age-related differences are numerically consistent with previously reported trends; together these indicate that any locally derived reference framework should remain sex- and age-stratified. The pattern we observed in ventricular volumes merits separate comment, because it has a recognised technical as well as physiological basis. Men had larger end-diastolic and stroke volumes than women, a difference that survived correction for multiple comparisons, whereas ejection fraction did not differ by sex at either rest or stress. This dissociation is expected if the underlying difference is one of chamber size rather than contractile performance, since ejection fraction is a ratio and is therefore relatively preserved when end-diastolic and end-systolic volumes scale together. It is also relevant that the measurement of small ventricles on gated imaging is subject to systematic overestimation of ejection fraction and underestimation of volume, an effect attributable to limited spatial resolution and demonstrated for small hearts on gated perfusion imaging [24]. Because women in this subgroup had the smaller ventricles, any residual small-heart effect would act in the direction of the observed findings, and we cannot exclude a technical contribution to the magnitude of the volume difference. The absence of a sex difference in ejection fraction, and the wide confidence intervals around the volume reference limits in women, both argue for caution in applying sex-specific volume limits derived from a subgroup of this size. A related consideration concerns the comparison of stress and rest ejection fraction. Gated images acquired after vasodilator stress are not acquired at peak hyperaemia, and the ejection fraction measured post-stress reflects a recovering rather than a maximally stressed ventricle; the determinants of the ejection fraction response to vasodilator stress in gated ^82^Rb imaging are accordingly complex and are influenced by haemodynamic as well as ischaemic factors [25]. We report stress and rest values separately for this reason and do not interpret the difference between them as an index of ischaemic burden. Readers applying these reference estimates should note that stress and rest limits are not interchangeable and should be used against measurements acquired under comparable conditions.

### 4.5. Clinical Application and Future Directions

Two practical implications follow from these findings. The first concerns how a quantitative PET service should establish its own interpretive limits. Our results indicate that both the central tendency and the dispersion of absolute flow in a locally defined normal group can differ from published series, and that the uncertainty around a percentile limit derived from a small group is substantial. A laboratory introducing quantitative reporting should therefore characterise its own pipeline in patients it considers normal, report the uncertainty around any limit it derives, and treat externally published thresholds as a starting point for comparison rather than as directly transferable values. The second concerns the design of the confirmatory work these estimates require. A study capable of establishing reference values for this population would need prospective recruitment of asymptomatic individuals at low pre-test probability, systematic recording of cardiometabolic risk factors and medication, objective exclusion of atherosclerosis by coronary calcium scoring or computed tomographic angiography, and a sample size sufficient for stable estimation of extreme percentiles within sex and age strata. The confidence intervals reported here provide a basis for that sample-size calculation. Until such data exist, the values we report are best used to indicate the range and behaviour of quantitative parameters in this setting rather than as diagnostic thresholds.

### 4.6. Prognostic Relevance of Reduced Flow Reserve

Although this analysis is descriptive, reduced MFR is among the most consistently validated prognostic markers in cardiac PET: noninvasive coronary flow reserve independently predicts cardiac death and adds risk information beyond perfusion and clinical variables [22], and integrated physiological assessment shows flow reserve to be a dominant correlate of cardiovascular mortality [26]. The prognostic behaviour of flow reserve in the present cohort is examined in a separate companion analysis and is not addressed here.

### 4.7. Limitations

Several limitations should be acknowledged. First, the clinically defined normal subgroup comprised clinically referred patients judged disease-free rather than prospectively screened healthy volunteers, and was modest in size (*n* = 44), so the tabulated centiles, particularly within age strata, are estimates that warrant confirmation in larger cohorts. Coronary calcium was documented in only a minority of reports, limiting a stricter calcium-zero definition. Exclusion of disease relied on relative perfusion assessment without universal coronary calcium scoring or invasive angiography, so the subgroup may include individuals with early or subclinical atherosclerosis or undetected microvascular dysfunction, which would bias the reference limits downward. These estimates should therefore be regarded as clinically defined rather than physiologically verified norms. Second, the cohort was assembled retrospectively from clinically indicated studies at a single centre, and the indications for referral will have shaped its composition in ways that cannot be reconstructed. Third, cardiovascular risk factors and medication data other than body mass index were not systematically recorded and could not be retrieved, which prevents adjustment for factors known to influence hyperaemic flow and limits the mechanistic interpretation of the lower absolute values observed. Fourth, all quantification was performed with a single software platform, and absolute values are known to depend on the kinetic model and implementation used [10]; the estimates reported here should not be assumed to transfer to other pipelines. Fifth, the reference subgroup is small, and the bootstrap confidence intervals reported alongside each limit make the resulting uncertainty explicit; several intervals around the ventricular volume limits are wide, and their lower bounds are not physiologically plausible, which is why we present these as exploratory estimates and advise against their clinical use without confirmation. Left ventricular volumes are reported in absolute terms because body surface area could not be derived from the available data, so the observed sex differences may partly reflect body size rather than intrinsic factors. Resting haemodynamics were unavailable, so the sex difference in resting MBF could not be adjusted for loading conditions. chamber size. Finally, no outcome data are reported here, so the present analysis characterises the distribution of quantitative parameters and cannot address their prognostic significance in this population.

## 5. Conclusions

This exploratory study provides sex- and age-stratified reference values for absolute ^82^Rb PET myocardial blood flow, flow reserve, and LV function in an Arabian Gulf population. The global MFR lower reference limit (2.06, bootstrap 95% CI 2.03–2.20) reflects the subgroup’s entry criterion and cannot validate the conventional threshold; absolute-flow values were lower than Western low-risk references, with sex-related differences in resting flow and age-related differences that were numerically consistent with previously reported trends. Population- and pipeline-specific reference ranges should be used for accurate clinical interpretation, and these descriptive values warrant prospective confirmation in larger, healthy screened cohorts. These data establish that quantitative Rb-82 parameters in this Gulf cohort differ in central tendency from published Western series while showing sex-related differences in resting flow and age-related differences that were numerically consistent with previously reported trends, and they quantify the uncertainty attaching to reference limits derived from a subgroup of this size. They do not establish diagnostic thresholds for this population, do not validate the conventional flow reserve cut-off, and do not identify the mechanism underlying the lower absolute values observed. Each of those questions requires the prospective, objectively screened cohort described above.

## Figures and Tables

**Table 1:** Cohort characteristics: full PET cohort and C1 reference subgroup.

Characteristic	Analytic PET Cohort (*n* = 292)	C1 Reference Subgroup (*n* = 44)
Female/male	126/166	25/19
Female (%)	43.2%	56.8%
Age, mean +/− SD (y)	63.1 +/− 12.1	59.9 +/− 11.3
Age range (y)	12–91	39–85
BMI, mean ± SD (kg/m^2^)	31.5 ± 5.6	32.3 ± 5.6
Obesity (BMI ≥ 30), *n* (%)	182 (62.3%)	32 (72.7%)

PET C1 = normal perfusion + normal global MFR (≥2.0). The reference subgroup additionally required normal resting LVEF and no documented cardiac history (*n* = 44). C3 (normal perfusion + reduced MFR) was excluded from the normal reference. BMI is available in 292 of 330 studies. Other risk-factor data were not systematically recorded in the imaging database and could not be retrieved retrospectively.

**Table 2:** Stress/rest MBF and MFR reference values by territory: PET C1 patients (*n* = 44).

Parameter	N	Mean	SD	5th %ile ^1^	95% CI	25th %ile	Median	95th %ile
Stress MBF (mL/min/g)								
LAD	44	2.674	0.564	1.983	1.51–2.08	2.240	2.650	3.674
LCX	44	2.878	0.623	2.000	1.70–2.15	2.395	2.935	3.856
RCA	44	3.261	0.863	1.924	1.67–2.43	2.645	3.255	4.702
Global	44	2.840	0.571	2.025	1.63–2.20	2.345	2.940	3.686
Rest MBF (mL/min/g)								
LAD	44	0.993	0.251	0.681	0.63–0.79	0.818	0.930	1.407
LCX	44	1.070	0.297	0.703	0.60–0.81	0.900	1.040	1.728
RCA	44	1.077	0.297	0.706	0.65–0.80	0.877	1.035	1.652
Global	44	1.038	0.267	0.691	0.67–0.78	0.860	1.000	1.469
MFR (stress/rest)								
LAD	44	2.728	0.606	2.046	1.99–2.12	2.245	2.565	3.694
LCX	44	2.770	0.691	1.964	1.75–2.10	2.198	2.580	3.825
RCA	44	3.101	0.798	2.030	1.82–2.25	2.515	3.005	4.585
Global	44	2.811	0.624	2.060	2.03–2.20	2.275	2.720	3.996

^1^ Values are the 5th percentile of the clinically defined normal subgroup, shown with bootstrap percentile 95% CI (2000 resamples). Because the subgroup was defined in part by global MFR ≥ 2.0, the MFR percentiles describe a preselected distribution and are not independently derived physiological thresholds. ASNC/SNMMI thresholds: MFR < 2.0 = abnormal [4,5].

**Table 3:** Sex-stratified MBF and MFR reference values: PET C1 patients.

Parameter	Sex	N	Mean	SD	5th %ile	95% CI	Median	95th %ile
Global Stress MBF (mL/min/g)	Female	25	2.894	0.597	2.138	2.00–2.33	3.000	3.902
Global Stress MBF (mL/min/g)	Male	19	2.769	0.544	1.975	1.57–2.19	2.910	3.379
Global Rest MBF (mL/min/g)	Female	25	1.130	0.284	0.800	0.76–0.92	1.070	1.642
Global Rest MBF (mL/min/g)	Male	19	0.916	0.189	0.679	0.67–0.74	0.900	1.211
Global MFR	Female	25	2.612	0.497	2.044	2.03–2.21	2.570	3.416
Global MFR	Male	19	3.073	0.687	2.159	2.15–2.65	2.980	4.219
LAD MFR	Female	25	2.474	0.431	2.032	1.98–2.10	2.350	3.264
LAD MFR	Male	19	3.062	0.651	2.192	2.12–2.40	3.150	4.213
LCX MFR	Female	25	2.586	0.586	1.966	1.91–2.15	2.440	3.592
LCX MFR	Male	19	3.013	0.757	1.972	1.72–2.44	2.910	3.887
RCA MFR	Female	25	2.961	0.705	2.034	2.01–2.34	2.990	4.288
RCA MFR	Male	19	3.285	0.891	2.195	1.79–2.63	3.120	4.756

**Table 4:** Age-stratified MBF and MFR reference values: clinically defined normal subgroup.

Age Group	Parameter	N	Mean	SD	5th %ile	Median	95th %ile
<60	Global Stress MBF	24	2.938	0.566	2.069	3.105	3.542
<60	Global MFR	24	2.920	0.671	2.043	2.865	3.995
≥60	Global Stress MBF	20	2.723	0.569	2.019	2.655	3.722
≥60	Global MFR	20	2.680	0.550	2.155	2.585	3.749

Age stratified into two broad bands owing to the size of the clinically defined normal subgroup; centiles are exploratory. Between-group comparisons (Mann–Whitney U): stress MBF *p* = 0.160, r = 0.25; MFR *p* = 0.300, r = 0.19; rest MBF *p* = 0.687. Neither flow parameter differed significantly by age group.

**Table 5:** Sex differences in LV functional parameters within the clinically defined normal subgroup (Mann–Whitney U, Bonferroni-adjusted).

Parameter	Median Men	Median Women	U	*p* (Bonf.)	r	d [95% CI]	Sig.
Stress LVEF (%)	67	67	248	0.810	0.044	+0.05 [−0.55, 0.65]	ns
Stress EDV (mL)	82	61	351	0.006	0.478	+0.88 [0.25, 1.50]	*
Stress ESV (mL)	23	21	300	0.144	0.261	+0.49 [−0.12, 1.09]	ns
Stress SV (mL)	52	41	370	0.001	0.556	+1.03 [0.39, 1.67]	*
Rest LVEF (%)	60	59	250	0.764	0.055	−0.01 [−0.61, 0.59]	ns
Rest EDV (mL)	64	51	348	0.008	0.463	+0.88 [0.25, 1.50]	ns †
Rest ESV (mL)	25	21	330	0.028	0.387	+0.86 [0.23, 1.48]	ns †
Rest SV (mL)	38	31	354	0.005	0.488	+0.78 [0.16, 1.40]	*

Values are medians. U = Mann–Whitney U statistic. r = rank-biserial correlation, r = (2U)/(n_1_n_2_) − 1. Cohen’s d is calculated with pooled standard deviation; positive values indicate higher values in men. * *p* < 0.00625 (Bonferroni-corrected threshold for eight comparisons). † Nominally significant at *p* < 0.05 but did not survive correction. EDV = end-diastolic volume; ESV = end-systolic volume; LVEF = left ventricular ejection fraction; SV = stroke volume.

**Table 6:** Age effects on LV functional parameters (Kruskal–Wallis, clinically defined normal subgroup, <60 years *n* = 24 vs. ≥60 years *n* = 20).

Parameter	Modality	H (df = 1)	*p*	eta2	Sig.
Stress LVEF (%)	PET	0.08	0.777	−0.022	ns
Stress EDV (mL)	PET	2.28	0.131	0.030	ns
Stress ESV (mL)	PET	0.41	0.524	−0.014	ns
Stress SV (mL)	PET	2.10	0.147	0.026	ns

η^2^ = rank eta-squared; negative values arise when H is less than the degrees of freedom and indicate no detectable effect. Given subgroup sizes of 20–24, confidence intervals for η^2^ were unstable and are not reported; effect sizes should be interpreted as descriptive. No parameter differed significantly by age group.

**Table 7:** Sex-stratified LV functional reference values: clinically defined normal subgroup (*n* = 44).

Parameter	Sex	N	Mean	SD	5th %ile	95% CI	Median	95th %ile
LV Functional: Stress								
LVEF (%)	Female	25	66.1	8.1	54.2	52.0–59.0	67.0	78.2
LVEF (%)	Male	19	66.5	7.8	52.9	52.0–61.0	67.0	75.6
EDV (mL)	Female	25	63.3	18.2	42.2	20.0–52.6	61.0	88.8
EDV (mL)	Male	19	82.7	26.7	53.4	48.0–65.8	82.0	144.2
ESV (mL)	Female	25	22.5	11.1	11.2	3.0–14.0	21.0	37.2
ESV (mL)	Male	19	28.3	12.8	12.0	12.0–20.1	23.0	50.9
SV (mL)	Female	25	40.8	9.5	26.4	17.0–35.0	41.0	56.8
SV (mL)	Male	19	54.5	17.0	35.6	32.0–43.9	52.0	88.7
LV Functional: Rest								
LVEF (%)	Female	25	60.3	7.1	52.2	51.0–54.4	59.0	68.8
LVEF (%)	Male	19	60.2	5.4	51.9	51.0–57.5	60.0	69.4
EDV (mL)	Female	25	50.7	15.1	28.6	15.0–41.2	51.0	74.0
EDV (mL)	Male	19	68.8	26.4	38.4	33.0–52.5	64.0	127.1
ESV (mL)	Female	25	19.8	6.2	13.2	3.0–15.2	21.0	26.8
ESV (mL)	Male	19	27.5	11.6	13.9	13.0–19.3	25.0	51.2
SV (mL)	Female	25	30.9	10.3	15.4	12.0–24.4	31.0	47.2
SV (mL)	Male	19	41.4	16.6	24.5	20.0–32.2	38.0	75.5

Values shown are the 5th and 95th percentiles of the clinically defined normal subgroup, with bootstrap percentile 95% CI.

**Table 8:** Age-stratified LV functional reference values: clinically defined normal subgroup (*n* = 44).

Age Group	Parameter	N	Mean	SD	5th %ile	95% CI	Median	95th %ile
<60	Stress LVEF (%)	24	66.7	9.0	52.1	52.0–59.0	67.5	80.7
<60	Stress SV (mL)	24	48.7	15.6	32.6	17.0–39.3	47.0	64.2
≥60	Stress LVEF (%)	20	65.8	6.6	55.0	54.0–61.7	67.0	75.0
≥60	Stress SV (mL)	20	44.4	13.6	31.7	25.0–35.5	41.5	60.4

## Data Availability

The data supporting the findings of this study are available from the corresponding author upon reasonable request.
